# Dropless Cataract Surgery: The Effect of Intracameral Phenylephrine/Ketorolac Infusion as a Single Agent on Postoperative Outcomes

**DOI:** 10.7759/cureus.62866

**Published:** 2024-06-21

**Authors:** Michael T Christensen, Connor S Davenport, Han Y Yin, Atalie C Thompson, Keith A Walter

**Affiliations:** 1 Medical School, Wake Forest School of Medicine, Winston-Salem, USA; 2 Surgical Ophthalmology, Eye Surgeons Associates, Bettendorf, USA; 3 Surgical Ophthalmology, Wake Forest School of Medicine, Winston-Salem, USA; 4 Surgical Ophthalmology, Milan Eye Center, Cumming, USA; 5 Surgical Ophthalmology/Geriatrics, Atrium Health Wake Forest Baptist Medical Center, Winston-Salem, USA; 6 Surgical Ophthalmology, Atrium Health Wake Forest Baptist Medical Center, Winston-Salem, USA

**Keywords:** eye surgery, postoperative pain management, phenylephrine/ketorolac, omidria, dropless, phacoemulsification cataract surgery

## Abstract

Objective

The objective of this study was to assess the feasibility of using an intracameral phenylephrine/ketorolac infusion during cataract surgery as a single agent to prevent postoperative pain, inflammation, and other complications.

Methods

A prospective, single-group feasibility study was conducted in which phenylephrine/ketorolac infusion was administered during cataract surgery and no perioperative topical drops were initially prescribed. Patients underwent optical coherence tomography, corrected distance visual acuity testing, and slit lamp biomicroscopy examination at perioperative visits, during which they also reported symptoms of pain, irritation, and/or photophobia. A goal adverse event (AE) rate was set at ≤5.0%.

Results

A total of 94 eyes (60 patients) were included in this study. The AE rate was 13.8% (13/94 eyes) with pain/irritation in eight eyes, cystoid macular edema (CME) in three eyes, and corneal edema in three eyes.

Conclusions

Based on an AE rate goal of ≤5.0%, using intraoperative, intracameral phenylephrine/ketorolac alone was not deemed a feasible alternative to current postoperative eye drop regimens in our clinical setting. However, a 13.8% AE rate is comparable to the rates of postoperative CME, corneal edema, pain, and irritation in the published literature. Thus, more research is needed to truly define this approach as inferior or non-inferior to the current standard of care.

## Introduction

Following cataract surgery, patients are traditionally prescribed multiple postoperative eye drops, including an antibiotic, a steroid, and/or a non-steroidal anti-inflammatory drug (NSAID). However, topical eye drops can be costly, and recent research has shown that patients have difficulty administering them correctly [1,2]. Our team sought new ways to reduce this burden while maintaining current safety standards. One way this has been done in other studies is by replacing the postoperative antibiotic eye drop with an intracameral antibiotic injection at the time of surgery [3]. This method has proven to be an effective and safe alternative to topical antibiotics for preventing postoperative endophthalmitis.

A combination phenylephrine/ketorolac 1.0%/0.3% infusion (Omidria, Rayner Surgical Inc, Worthing, United Kingdom) recently received FDA approval to decrease postoperative pain and inflammation and maintain pupillary dilation during cataract surgery [4]. Because it was shown to almost completely inhibit prostaglandin production for greater than 10 hours in an animal study [5], we hypothesized that analogous to intracameral antibiotics replacing topical antibiotic eye drops, intracameral phenylephrine/ketorolac could supplant patient self-administered NSAIDs and steroid eye drops after cataract extraction.

## Materials and methods

This was a prospective, interventional, single-group feasibility study conducted at Atrium Health Wake Forest Baptist Hospital in Winston-Salem, North Carolina, United States, on patients undergoing cataract surgery between July 30, 2019, and June 17, 2021. As a feasibility study, there was no control group or randomization process. This study was approved by the Institutional Review Board of Atrium Health Wake Forest Baptist (approval number: 00050415). This trial was registered on ClinicalTrials.gov on March 6, 2019 (registration number: NCT03864133). All tenets of the Declaration of Helsinki were followed. Data collection and reporting followed all Health Insurance Portability and Accountability Act regulations. All participants signed written informed consent.

Patients were included in the study if they were undergoing routine elective cataract surgery and met the following inclusion criteria: age 55-90 years with significant visual cataracts in one or both eyes and able to tolerate outpatient cataract surgery under local anesthesia via either phacoemulsification and/or femtosecond assisted cataract surgery. Patients with well-controlled diabetes or hypertension were included. Exclusion criteria were the following: allergy to phenylephrine or NSAIDs, inability to sit steady and upright for optical coherence tomography (OCT), complications during surgery including posterior capsular rupture, vitreous loss, zonular dialysis, iris trauma, retained lens fragment, central macular thickness above 300 μm at baseline, current prostaglandin analog use, presence of an epiretinal membrane on preoperative OCT, inability to return for follow appointments, and patients who were pregnant, lactating, or planning to become pregnant.

Intraoperative management

Each procedure and perioperative visit was performed by one of the two total surgeons. After the initial incision, each patient received 0.33 mL intracameral phenylephrine/ketorolac 1.0%/0.3%. Following this dose, they received a continuous infusion of phenylephrine/ketorolac 1.0%/0.3% during the remainder of the operation to maintain their intraocular pressure. The rate of drug infusion varied based on the patient’s intraocular pressure and the length of surgery. After intraocular lens implantation, 0.5 mL of a 50% moxifloxacin solution was injected intracamerally.

Postoperative management

No topical drops were initially prescribed in the postoperative period. All patients participating in the study received macular scans via OCT at preoperative (pre-op), two-week (POW2), and six-week (POW6) follow-up visits to record baseline and postoperative central macular thickness measurements. At postoperative day one (POD1), POW2, and POW6 visits, patients received anterior chamber (AC) cell grading, which was determined to be trace, 1+, or 2+ based on the clinician’s examination using slit lamp biomicroscopy (Table 1). Corrected distance visual acuity (CDVA) was assessed at all four visits using the logarithm of the minimum angle of resolution (logMAR) chart and scale. Patients' age and sex were also recorded.

**Table 1:** AC cell grading by approximate number of cells seen in the AC via a 1 mm^3 slit lamp examination AC: anterior chamber

Approximate number of cells seen in the AC	AC Cell Grading
<10 cells	Trace
10-20 cells	1+
>20 cells	2+

Patients subjectively reported their postoperative pain and/or irritation during the review of systems at postoperative follow-up visits to both technician and physician. Pain and/or irritation symptoms also included photophobia. Cystoid macular edema (CME) was diagnosed based on evidence of increased central macular thickness on macular OCT at the two and six-week postoperative scans. Early postoperative corneal edema was determined based on the physician’s clinical exam using slit lamp biomicroscopy and a review of OCT of the macula for intraretinal cysts and central macular thickening.

Statistical analyses

Statistical analyses included the calculation of patient mean age, logMAR score, AC cell grading estimates, and central macular thickness on OCT. In addition, the proportion of AEs, including pain/irritation, CME, or early postoperative corneal edema, was measured. An a priori AE rate goal of ≤5.0% was set. While the recruitment goal was 200 eyes, the plan was to terminate the study if the AE rate exceeded 5.0% after 100 eyes.

## Results

A total of 94 eyes (60 patients) were included in this study after six eyes were excluded for not meeting the study criteria. Of the 94 remaining eyes, 47 (50%) self-identified as male and 47 (50%) identified as female. The mean patient age was 69.67 years with a standard deviation (SD) of 6.96 years.

At postoperative follow-up visits, eight eyes (8.5%) reported pain, irritation, and/or photophobia and were treated with a combination of the following topical eye drops, depending on symptoms, their duration, and the physician seen at follow-up visits: bromfenac, fluorometholone, prednisolone, polymyxin, and moxifloxacin. An additional three eyes (3.2%) developed postoperative CME and were treated with topical bromfenac and/or prednisolone drops. Lastly, three eyes (3.2%) developed early postoperative corneal edema and were treated with topical fluorometholone and/or bromfenac drops. One patient with corneal edema also reported irritation and is included in both categories. Overall, 13 eyes (13.8%) experienced an AE.

The quantity of phenylephrine/ketorolac intracameral infusion during surgery was documented for 78 eyes (81.3%) and ranged between 40 and 200 mL. The average patient received approximately 101 mL. Of the 13 eyes with a documented AE, 10 had their phenylephrine/ketorolac infusion amounts documented and received an average of 112 mL.

All patients attended pre-op and POD1 visits. Three patients (three eyes, 3.2%) did not attend their POW2 visits, and 15 patients (16 eyes, 17.0%) did not attend their POW6 appointments. Nine eyes (9.6%) either did not receive OCT scans or had poor-quality OCTs at their pre-op visits, and consequently, the central macular thickness could not be measured. Similarly, 18 eyes (19.1%) at their POW2 visits and 12 eyes (12.6%) at their POW6 visits either did not receive OCT scans or had poor quality OCTs and were not included in the result data. All available logMAR, AC Cell, and central macular thickness measurements at these visits are detailed in Table 2.

**Table 2:** Perioperative measurements Displays mean corrected distance logMAR visual acuity scores, anterior chamber cell gradings, and central macular thicknesses at perioperative appointment visits with standard deviations. AC: anterior chamber; logMAR: logarithm of the minimum angle of resolution; Pre-op: preoperative; POD1: postoperative day 1; POW2: postoperative week 2; POW6: postoperative week 6; SD: standard deviation

	Pre-op	POD1	POW2	POW6
logMAR, mean±SD	0.2838 ± 0.23	0.3336 ± 0.29	0.1802 ± 0.18	0.1156 ± 0.16
AC Cell, mean±SD	N/A	0.7819 ± 0.66	0.2747 ± 0.54	0.0790 ± 0.22
Central Macular Thickness, mean±SD	260.58 ± 22.66 μm	N/A	272.23 ± 27.09 μm	284.15 ± 32.00 μm

## Discussion

In this study, examining intracameral infusion of a phenylephrine/ketorolac drug, the rate of postoperative AEs (13.8%) exceeded the acceptable threshold of ≤5.0%, so enrollment was terminated early. Based on this AE rate and the study’s early termination, we have found that phenylephrine/ketorolac is not a feasible alternative to standard perioperative topical regimens. The AE goal rate was determined based on our prior study in the same clinical setting [6], which achieved a lower rate of CME of approximately 0.09% (1/1090 eyes) in a cohort using preoperative and postoperative bromfenac alone. Rates of postoperative pain, irritation, and early postoperative corneal edema were not recorded, but the combined AE rate was presumed to be ≤5.0%. However, in the phase three FDA approval studies, 172 out of 403 (43.9%) patients who received phenylephrine/ketorolac experienced an eye disorder as compared to 177 out of 405 (43.7%) patients who received placebo [4]. This may suggest that the goal set for this study (≤5.0%) was too conservative, and a higher AE threshold could have been used.

Studies report that the estimated incidence of postoperative pain and irritation using multi-drop regimens is between 1.72% [7] and 10.0% [8] after phacoemulsification and intraocular lens placement. Thus, a rate of 8.5% of eyes with pain, irritation, or photophobia after treatment with intracameral phenylephrine/ketorolac could be within acceptable limits. One limitation of this study was the subjective nature of patient-reported symptoms. In a future project, it may be helpful to rigorously define the parameters for patient pain, irritation, and photophobia using standardized scales.

Rates of postoperative CME (3.2%) and early postoperative corneal edema (3.2%) in this study were also similar to reported incidences in other studies where patients were treated with multi-drop regimens. Most studies estimate the incidences to be between 0.09% [6] and 12.0% [9] for CME [10-13] and 0.87% [14] and 11.3% [15] for early postoperative corneal edema. This suggests that intraoperative phenylephrine/ketorolac administration may not be an inferior practice to prevent CME and corneal edema when compared with literature rates. This finding further supports the study of this treatment modality as a possible alternative to standard perioperative care. Moreover, it was previously shown that postoperative steroid drop use may not reduce the likelihood of postoperative CME [6], inferring that cataract surgeons who routinely prescribe steroid eye drops may be unnecessarily exposing their patients to known side effects without any additional benefit of CME risk reduction.

This study had several strengths. Notably, because it was a pragmatic feasibility study, this study focused on establishing a baseline rate of AEs (i.e. careful documentation of signs of iritis, CME, early postoperative corneal edema, etc.) for this treatment regimen, which enables other surgeons and researchers to assess this study’s results and compare them to their own patient cohorts. These results appear to be comparable to rates of AEs in literature but did not require a lengthy postoperative topical drops course or tapering schedule which can be confusing, expensive, and burdensome for patients. This may suggest that intracameral administration of phenylephrine/ketorolac alone could be a more practical approach to managing patients following cataract surgery. With additional research, appropriate treatment with this drug alone may prove to be particularly useful for clinicians whose patients experience difficulty self-administering topical eye drops (i.e. patients with arthritis or Parkinson’s disease), have a history of poor adherence to eye drop regimens (i.e. patients with dementia or patients dependent on family members to administer their medications), or cannot afford postoperative medications.

At the same time, there are some limitations too. As a feasibility study, there was no randomization process or control group. Thus, one prominent limitation is that no comparative statements can be made about the significance of the postoperative measurements or rate of AEs. Additionally, without a control arm, we cannot confidently conclude that our results were not affected by confounding factors present within our patient population, the amount of phenylephrine/ketorolac infused, or the care patients received in our department. As a result, we cannot recommend this method be adopted in the clinical setting until a randomized controlled trial is performed which establishes non-inferiority. Finally, a sample size of 94 eyes may also limit our ability to draw strong conclusions from the data, but no sample size calculations could be conducted without a control group.

Further research to improve the care of patients who undergo cataract surgery could explore the use of perioperative oral NSAID tablets in combination with intracameral phenylephrine/ketorolac infusion and compare results to a control cohort undergoing usual postoperative management. A study like this may better identify the most effective approach to avoid postoperative AEs while limiting the misuse and financial burden incurred with topical drops. Alternatively, some studies have shown that intravitreal injections, whether trans-zonular [1] or via the pars plana [16], may improve treatment outcomes when compared with the commonly accepted postoperative multi-drop topical regimen.

## Conclusions

Based on an AE rate goal of ≤5.0%, using intraoperative, intracameral phenylephrine/ketorolac alone was not deemed a feasible alternative to current postoperative eye drop regimens in our clinical setting. However, a 13.8% AE rate is comparable to the rates of postoperative CME, corneal edema, pain, and irritation in the published literature. Thus, more research is needed to truly define this approach as inferior or non-inferior to the current standard of care.
